# Changes in type 2 innate lymphoid cells and serum cytokines in sublingual immunotherapy in pediatric patients with allergic rhinitis

**DOI:** 10.1186/s12887-022-03788-z

**Published:** 2023-01-09

**Authors:** Xiaoqiang Wang, Yang Shen, Suling Hong, Houyong Kang, Xia Ke

**Affiliations:** Department of Otolaryngology-Head and Neck Surgery, The First Affiliated Hospital of Chongqing Medical University, No. 1 Youyi Road, Yuzhong District, Chongqing, 400016 People’s Republic of China

**Keywords:** Sublingual immunotherapy, Allergic rhinitis, Type 2 innate lymphocytes, Pediatric patients

## Abstract

**Background:**

Type 2 innate lymphoid cells (ILC2) are upregulated in childhood allergic rhinitis (AR) and are associated with AR severity. This study aimed to investigate changes in the ILC2 milieu in pediatric patients with AR after sublingual immunotherapy (SLIT).

**Methods:**

Forty- pediatric patients with AR received house dust mite (HDM) allergen extract for SLIT group and thirty pediatric patients received placebo in the study, respectively. The levels of ILC2, ILC2-related cytokines (IL-5/IL-13) and their transcription factors (GATA binding protein 3, retinoic acid-related orphan receptor α) in the circulation were assessed after 1- and 2-year SLIT. Moreover, peripheral blood mononuclear cells (PBMCs) in patients were prepared and stimulated by recombinant thymic stromal lymphopoietin, IL-25, and IL-33 after 2-year SLIT. Subsequently, the levels of ILC2, IL-5, and IL-13 were tested.

**Results:**

The frequency of ILC2 and the levels of their transcription factors in the circulation were significantly decreased after SLIT in the SLIT group. The levels of ILC2-related cytokines in the SLIT group showed the same trend. The frequency of ILC2 was positively correlated with transcription factors and cytokines after SLIT. SLIT was observed to reduce the ability of HDM sensitization to generate the ILC2 milieu in PBMCs.

**Conclusions:**

Changes in the ILC2 milieu may be correlated with the curative effect and immune regulation function of SLIT. Our results suggested that the regulatory effect on ILC2 is part of the therapeutic mechanism of SLIT.

**Supplementary Information:**

The online version contains supplementary material available at 10.1186/s12887-022-03788-z.

## Background

Allergic rhinitis (AR), which is an allergic immunological disease of the nasal mucosa, has become a global health problem that affects both adults and children [1, 2]. Studies have shown that the prevalence of AR is rapidly increasing in Chinese children, and the treatment of children’s AR has become the focus of current studies in our country [3]. Although traditional treatment methods (such as nasal glucocorticoid, anti-leukotriene drugs) can achieve certain effects, they cannot modify the immune disorder in AR. In recent years, sublingual immunotherapy (SLIT), a type of specific immunotherapy, is believed to be involved in the regulation of immune disorders in AR by correcting Th2-biased reaction and could treat AR from the source of the disease [4].

Type 2 innate lymphoid cells (ILC2), a member of the innate lymphoid cells, are believed to be involved in the pathogenesis of AR [5, 6]. Although there is a lack ofadaptiveimmunecells(T, B cells) or other granulocyte monocyte lineage markers, they can play similar role as Th2 cells. For example, it can proliferate and produce Th2 cytokines (interleukin [IL]-5/IL- 13) in the presence of thymic stromal lymphopoietin (TSLP), IL-25, and IL-33, which could induce high eosinophil expression and AR onset [7, 8]. There have been many studies on the correlation between ILC2 and AR or its treatment [9]; however, changes in the ILC2 milieu during SLIT have not been established. According to the results of previous studies that house dust mites (HDMs) can promote the abnormally high ILC2 expression and the mature application of HDM-specific SLIT in China [6, 10, 11], the present study aimed to investigate the changes in ILC2 milieu-related indicators in Chinese pediatric patients with HDM-AR during SLIT to clarify the correlation between ILC2 and SLIT treatment mechanisms and provide evidence for the theoretical system of the SLIT mechanism.

## Methods

### Patients

The present study enrolled 70 children with AR who were allergic to HDMs only, and all pediatric patients had a history of allergy for at least 2 years. The age range of children included in the study was 6–10 years. The criteria of the Initiative on Allergic Rhinitis and its Impact on Asthma were used for disease diagnosis [12]. That is, patients with AR should presented with a characteristic history of watery nasal discharge, nasal obstruction, sneezing, itching in the nose, were positive for IgE specific to antigens such as HDM. The skin prick test (SPT) or serum immunoglobulin E (IgE) specific to common inhalant allergens (dust mites, pets, molds, cockroaches) was used to identify allergic status. Wheal diameter ≥ 2 mm in the SPT test or the detection value of specific IgE greater than 3.5 IU/mL was considered a positive result. The exclusion criteria of the present study were as follows: patients with chronic diseases of the heart, lung, and digestive system and other chronic diseases (asthma, malnutrition, cystic fibrosis, drug-induced rhinitis, occupational rhinitis, or any complications) and those with a long history of corticosteroid, antihistamine, or leukotriene receptor antagonist use [1]. The Code of Ethics of the World Medical Association (Declaration of Helsinki) for experiments involving humans was the criterion used in the present study. This study was approved by the Ethical Committee of Chongqing Medical University, and informed consent was obtained from all subjects before being selected in this study.

### Administration of immunotherapy

All subjects were randomly divided into the SLIT group (*n* = 40) and control group (*n* = 30). For immunotherapy, HDM allergen extract (CHANLLERNGEN, Wolwo Pharma Biotechnology Company, Zhejiang, China) was used. The treatment process consisted of two stages: Dermatophagoidesfarinae drops Nos. 1, 2, and 3 were used for initiation therapy, and drops Nos.4 and 5 were used for maintenance therapy. The processes were as follows: (1) During the initiation therapy (up-dosing phase), pediatric patients received increasing doses of HDM allergen extract under the guidance of their parents (No. 1, 1 mg/mL; No. 2, 10 mg/mL; No. 3, 100 mg/mL) for 3 weeks at first. (2) During the maintenance therapy, pediatric patients received three drops once daily (No. 4, 333 mg/mL; No. 5, 1000 mg/mL) since week 4. The controls received glycerin saline solution (50 mg/mL), which consisted of 50% saline buffer and 50% glycerol [1]. The drops under the tongue were dropped and swallowed after maintaining for 2–3 min. Importantly, each subject should have a treatment period of at least 2 years, and all subjects received symptomatic drugs only when symptoms appeared.

### Assessment of disease severity

The severity of clinical symptoms of AR was assessed using the Total 5 Symptom Score (T5SS) [6]. According to typical AR symptoms, such as itchy nose, stuffy nose, rhinorrhea, sneezing, and itchy eyes, a 0–3 point system was used for assessment (0, asymptomatic; 1, mild [symptomatic but not annoying]; 2, moderate [annoying but tolerable symptoms]; 3, serious [annoying and intolerable symptoms]). During the 2-year SLIT period, the patient’s parents were required to help complete the daily recording of symptom scores.

### Sample collection

The present study collected 10 mL of fasting venous blood samples from each subject from 6 a.m. to 8 a.m. using heparin anticoagulant tubes. The blood samples were then divided into two parts: one part was centrifuged for 15 min (3000 g, 4 °C) and stored at − 80 °C for further analysis; the other part was used for preparation of peripheral blood mononuclear cells (PBMCs) to complete further detection. Moreover, the electrochemiluminescence immunoassay method was used to measure eosinophil cationic protein (ECP) in the subjects’ serum. The present study completed nasal lavage using the method described in a previous study [13]. Briefly, 0.9% NaCl solution (5 mL/nostril) was provided to each subject to lavage the nasal cavity, the samples were centrifuged, and the supernatants were stored at − 80 °C for cytokine detection. Blood and nasal samples were collected at 0, 1, and 2 years for detection.

### Preparation of PBMCs

Blood samples were collected at 0, 1, and 2 years after SLIT. Lymphoprep (Fresenius Kabi Norge AS, Oslo, Norway) was used for the preparation of PBMCs. The standard operation was performed according to the manufacturer’s instructions. The sorted cells were divided into two parts: one part (PBMCs from all samples at 0, 1, and 2 years after SLIT) was prepared for flow cytometry directly; the other part (PBMC from all samples at 2 years after SLIT) was used for the intervention trial.

### Flow cytometry

To observe the level of ILC2 in subjects, RPMI- 1640, which contained streptomycin, 5% human AB serum, penicillin, and glutamine (5 mmol/L) solution, was added to 24-well plates, and the isolated PBMCs were cultured at a concentration of 2 × 10^6^/mL. Subsequently, phorbol myristate acetate (PMA) (50 ng/mL) and ionomycin (500 ng/mL, both from Sigma-Aldrich) were used for PBMC stimulation for 4 h; finally, brefeldin A (BD, Oxford, United Kingdom) was used for.

culture for 3 h. The lineage cocktail method was used to sort ILC2s according to a method described elsewhere [14]. In short, a cell lineage cocktail that could completely exclude dendritic cells, adaptiveimmunecells (T, B cells), mast cells, basophils, monocytes, and hematopoietic progenitor cells was used. Subsequently, Lin^−^CRTH2^+^CD127^+^ lymphocytes were marked and sorted out as ILC2s [15]. Flow cytometry was performed using a FACScan flow cytometer (BD Biosciences). FACSDiva software (BD Biosciences) was used to analyze the data, and the percentage of PBMCs was used to represent the frequency of ILC2s.

### Real-time polymerase chain reaction for transcription factors

TRIzol reagent (Life Technologies, Carlsbad, CA, USA) was used to extract total RNA from PBMCs. cDNA was synthesized using the cDNA kit (Qiagen). SYBR Green Universal PCR Master Mix (Bio-Rad, Hercules, CA, USA) was used for PCR amplification. The results were normalized to glyceraldehyde 3-phosphate dehydrogenase (GAPDH). The primers for GATA binding protein 3 (GATA3), retinoic acid-related orphan receptor α (RORα), and GAPDH were as follows: GATA3 [sense, 5′-AGG CTC GGG AAA GAG GTG ACA-3′; antisense, 5′-GGC TCC TGC CAA TTC ATT CG-3′], RORα [sense, 5′-AAA CAA GCA GCG GGA GGT GA-3′; antisense, 5′-TGG CAA ACT CCA CCA CAT AC-3′], and GAPDH [sense, 5′-AGC CAC ATC GCT CAG ACAC-3′; antisense, 5′-GCC CAA TAC GAC CAA ATCC-3′] [16, 17]. The gene expression data related to the housekeeper gene GAPDH were analyzed using the 2^-△△CT^ method.

### Enzyme-linked immunosorbent assay (ELISA) for the protein expression of ILC2-related cytokines

The levels of cytokines (IL-5/IL- 13) in serum and nasal lavage were detected using the enzyme-linked immunosorbent assay (ELISA) kits (R&D Systems, Minneapolis, MN, USA) according to the manufacturer’s protocol. The detection sensitivities of the assays were as follows: IL-5, 7.8 pg/mL; IL- 13, 125 pg/mL.

### Cell intervention and flow cytometry

PBMCs from blood samples collected at 2 years after SLIT were cultured in 1 mL medium (2 × 10^6^ cells/ml) added with PMA (50 ng/mL), and ionomycin (500 ng/mL) (Sigma-Aldrich, St. Louis, MO, USA) was used for culture of PBMCs in 5% CO2 at 37 °C for 5 days in the presence or absence of HDM extract (20 μg/mL HDM, RayBiotech, USA), IL-2 (500 ng/mL, PeproTech,

Rocky Hill, NJ, USA), TSLP (500 ng/mL, PeproTech, Rocky Hill, NJ, USA), IL-33 (500 ng/mL, R&D, Minneapolis, MN, USA), and IL-25 (500 ng/mL, R&D, Minneapolis, MN, USA) [1, 15]. The trypan blue exclusion test was used to assess the viability of the cultured cells. The results showed that less than 5% of the cultured cells died, indicating that cell viability was high. The complete detection of ILC2 frequency by flow cytometry was consistent with the previously described method in the present study.

### ELISA for protein expression of ILC2-related cytokines after intervention

After 5 days of culture, the supernatant from the cells after the intervention was collected and stored at − 8 0 °C until use according to the previously described method in the present study. ELISA kits (R&D Systems, USA) were used to detect the protein expression of ILC2-related cytokines after intervention.

### Statistical analyses

Means ± standard error of the means were used to represent data. For comparison, the nonparametric Mann-Whitney U test and t-test were used in the present study. All statistical analyses were performed using the Statistical Product and Service Solutions version 22.0 software. Spearman rank correlation analysis was used to assess the correlations between the different indicators. The significance level was set at *P* < 0.05.

## Results

### Demographic characteristics of subjects and clinical efficacy of SLIT

The demographic characteristics of the patients at 0 month after SLIT (baseline) are shown in Table 1. At the final time of the present study, the completion rates of the 2 - year course of treatment were 92.5% (37) and 93.3% (28) in the SLIT and control groups, respectively. Data from the present study showed that the symptom scores in the SLIT group were significantly lower compared with those in the control group (*P* < 0.05) (Table 1).

**Table 1:** Demographic characteristic of patients with AR

	SLIT group	Control croup	*P* values
Number	40	30	
Sex (male/female)	21/19	17/13	
Age (years)	8.1 ± 1.3	7.9 ± 1.1	0.621
Coexisting allergic diseases	0/40	0/30	
Specific IgE (kU/l)			
HDM	47 ± 3.6	45 ± 3.2	
Animal danders	<0.35	<0.35	
Cockroach	<0.35	<0.35	
SPT positive	40/40	30/30	
Baseline symptoms (T5SS)	8.2 ± 1.4	8 ± 1.4	0.702
T5SS after 1-year SLIT	4 ± 1.3^*^	7.9 ± 1.4	<0.05
T5SS after 2-year SLIT	2.1 ± 0.7^**^	7.7 ± 1.3	<0.05

*Compared with baseline, *P* < 0.05

**Compared with score after 1-year SLIT, *P* < 0.05

*AR* allergic rhinitis; *SLIT* sublingual immunotherapy

### Decreased ILC2 frequency in PBMC during SLIT treatment

**Fig. 1:**
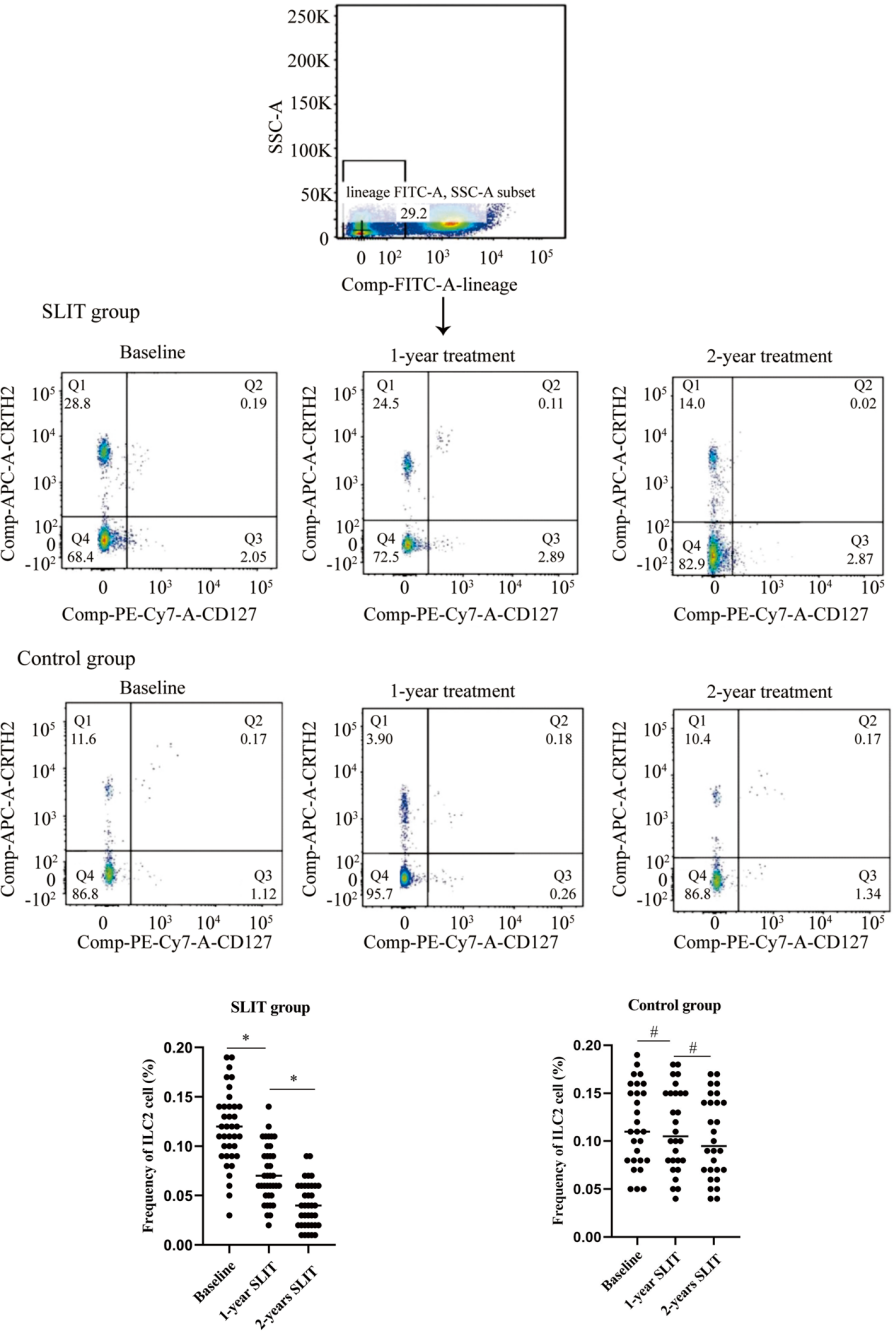
After completing the 1- and 2-year treatment period, the significant downregulation of ILC2 frequency in PBMCs from AR donor. *P < 0.05, #*P* > 0.05. ILC2, type 2 innate lymphoid cells; PBMCs, peripheral blood mononuclear cells; AR, allergic rhinitis

There was no significant difference in the level of ILC2 between the SLIT group and the control group at the baseline period (0.118 ± 0.036 vs 0.116 ± 0.042, *P*>0.05). After completing the 1-year treatment period, the frequency of ILC2 in the SLIT group decreased significantly (0.118 ± 0.036 vs 0.072 ± 0.029, *P*<0.05), and a similar trend was maintained after the completion of the 2-year treatment period (0.072 ± 0.029 vs 0.041 ± 0.022, *P*<0.05) (Fig. 1). However, the present study failed to observe a significant decrease in the frequency of ILC2 cells in the control group after 1- year treatment (0.116 ± 0.042 vs 0.113 ± 0.043, *P* = 0.733) and 2-year treatment (0.113 ± 0.043 vs 0.103 ± 0.042, *P* = 0.438) (Fig. 1).

### Decreased transcription factor mRNA levels during SLIT treatment

Compared to the baseline level, mRNA levels of ILC2-related transcription factors (GATA3, RORα) were significantly lower in the SLIT group after the completion of the 1-year treatment (GATA3: 2.82 ± 0.47 vs 2.20 ± 0.55, RORα: 3.12 ± 0.61 vs 2.20 ± 0.64, *P*<0.05); the mRNA levels in the SLIT group further decreased significantly after the completion of the 2-year treatment (GATA3: 2.20 ± 0.55 vs 1.59 ± 0.52, RORα: 2.20 ± 0.64 vs 1.47 ± 0.55, *P*<0.05)(Fig. S1). However, there were no significant changes in the mRNA levels of ILC2-related transcription factors in the control group after 1-year treatment (GATA3: 2.71 ± 0.48 vs 2.66 ± 0.47, *P*_GATA3_ = 0.677; RORα: 2.97 ± 0.70 vs 2.89 ± 0.70, *P*_RORα_ = 0.707) and 2-year treatment (2.66 ± 0.47 vs 2.63 ± 0.46, *P*_GATA3_ = 0.806; 2.89 ± 0.70 vs 2.80 ± 0.68, *P*_RORα_ = 0.628) (Fig. S1).

### Decreased serum and nasal inflammatory cytokine protein levels during SLIT treatment

Compared to baseline levels (IL-5_nasal_: 34.73 ± 7.91 pg/mL, IL-5_serum_: 63.44 ± 12.71 pg/mL; IL-13_nasal_: 122.85 ± 10.51 pg/mL, IL-13_serum_: 154.59 ± 21.49 pg/mL), the levels of IL-5 and IL-13 in the nasal (IL-5: 22.82 ± 5.76 pg/mL, IL-13: 90.80 ± 11.41 pg/mL) and serum (IL-5: 37.11 ± 9.64 pg/mL, IL-13: 119.57 ± 23.27 pg/mL) were significantly lower in the SLIT group after the completion of the 1-year treatment (*P*<0.05); the protein levels in the SLIT group further decreased significantly after the completion of the 2-year treatment (IL-5_nasal_: 15.24 ± 3.96 pg/mL, IL-5_serum_: 26.63 ± 7.66 pg/mL; IL-13_nasal_: 67.14 ± 13.11 pg/mL, IL-13_serum_: 94.36 ± 23.15 pg/mL; *P*<0.05) (Fig. S2). The levels of the same indicators in the control group did not change significantly after 1- year treatment (IL-5_nasal_: 34.67 ± 1.94 pg/mL vs 32.69 ± 7.19 pg/mL, *P* = 0.301; IL-5_serum_: 64.10 ± 11.32 pg/mL vs 62.30 ± 11.01 pg/mL, *P* = 0.550; IL-13_nasal_: 119.83 ± 13.17 pg/mL vs 117.11 ± 12.95 pg/mL, *P* = 0.440; IL-13_serum_: 151.25 ± 21.77 pg/mL vs 146.92 ± 21.16 pg/mL, *P* = 0.453) and 2-year treatment (IL-5_nasal_: 32.69 ± 7.19 pg/mL vs 30.64 ± 7.27 pg/mL, *P* = 0.453; IL-5_serum_: 62.30 ± 11.01 pg/mL vs 60.61 ± 10.56 pg/mL, *P* = 0.560; IL-13_nasal_: 117.11 ± 12.95 pg/mL vs 114.61 ± 13.15 pg/mL, *P* = 0.560; IL-13_serum_: 146.92 ± 21.16 pg/mL vs 143.27 ± 21.31 pg/mL, *P* = 0.522) (Fig. S2). The present study also observed a significant decline in nasal ECP in the SLIT group after 1-year treatment (24.40 ± 3.99 ng/mL vs 16.01 ± 3.21 ng/mL, *P*<0.05) and 2-year treatment (16.01 ± 3.21 ng/mL vs 8.25 ± 2.50 ng/mL, *P*<0.05); nevertheless, the SLIT treatment did not affect the level of serum ECP in the SLIT group (29.92 ± 7.24 ng/mL vs 28.74 ± 7.12 ng/mL vs 27.80 ± 6.76 ng/mL; *P*_1-year_ = 0.481, *P*_2-year_ = 0.560) (Fig. S3). Both serum and nasal ECP levels in the control group did not change after 1- and 2-year treatment (Serum: 30.30 ± 7.68 ng/mL vs 29.27 ± 7.72 ng/mL vs 28.08 ± 6.70 ng/mL; *P*_1-year_ = 0.619, *P*_2-year_ = 0.541. Nasal: 23.21 ± 3.93 ng/mL vs 22.10 ± 3.46 ng/mL vs 21.04 ± 3.46 ng/mL; *P*_1-year_ = 0.268, *P*_2-year_ = 0.258) (Fig. S3).

### Correlation analysis between inflammatory cytokine and transcription factor levels and frequency of ILC2 in the SLIT group

**Fig. 2:**
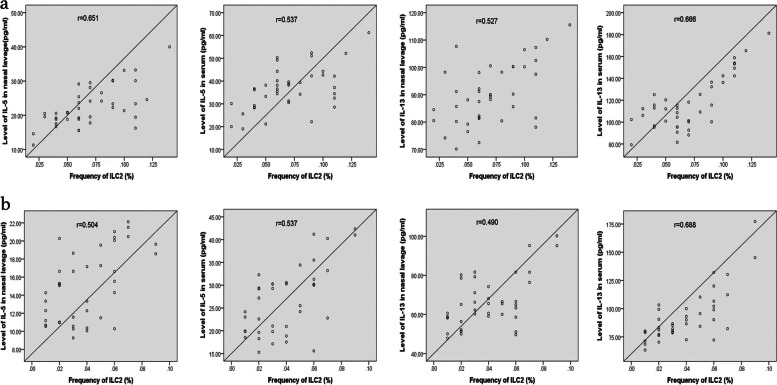
Positive correlations between the levels of inflammatory cytokines (IL-5/IL-13) and ILC2 frequency in the SLIT group after 1- and 2-year treatment. **a**: After 1-year treatment; (**b**): After 2-year treatment. IL, interleukin; ILC2; type 2 innate lymphoid cells; SLIT, sublingual immunotherapy

The present study observed positive correlations between the nasal or serum levels of IL-5, IL-13, and ILC2 frequency in the SLIT group after 1- and 2-year treatment (*P* < 0.01) (Fig. 2). The transcription factor levels (GATA3, RORα) were positively correlated with ILC2 frequency in the SLIT group after 1- and 2-year treatment (*P* < 0.01) (Fig. S4). In addition, the same trend was found between the nasal level of ECP, T5SS score, and ILC2 frequency in the SLIT group after 1- and 2-year treatment (*P* < 0.01) (Fig. S5).

### Decreased ILC2 milieu activation ability of PBMCs induced by HDM and stimulating factors after SLIT

**Fig. 3:**
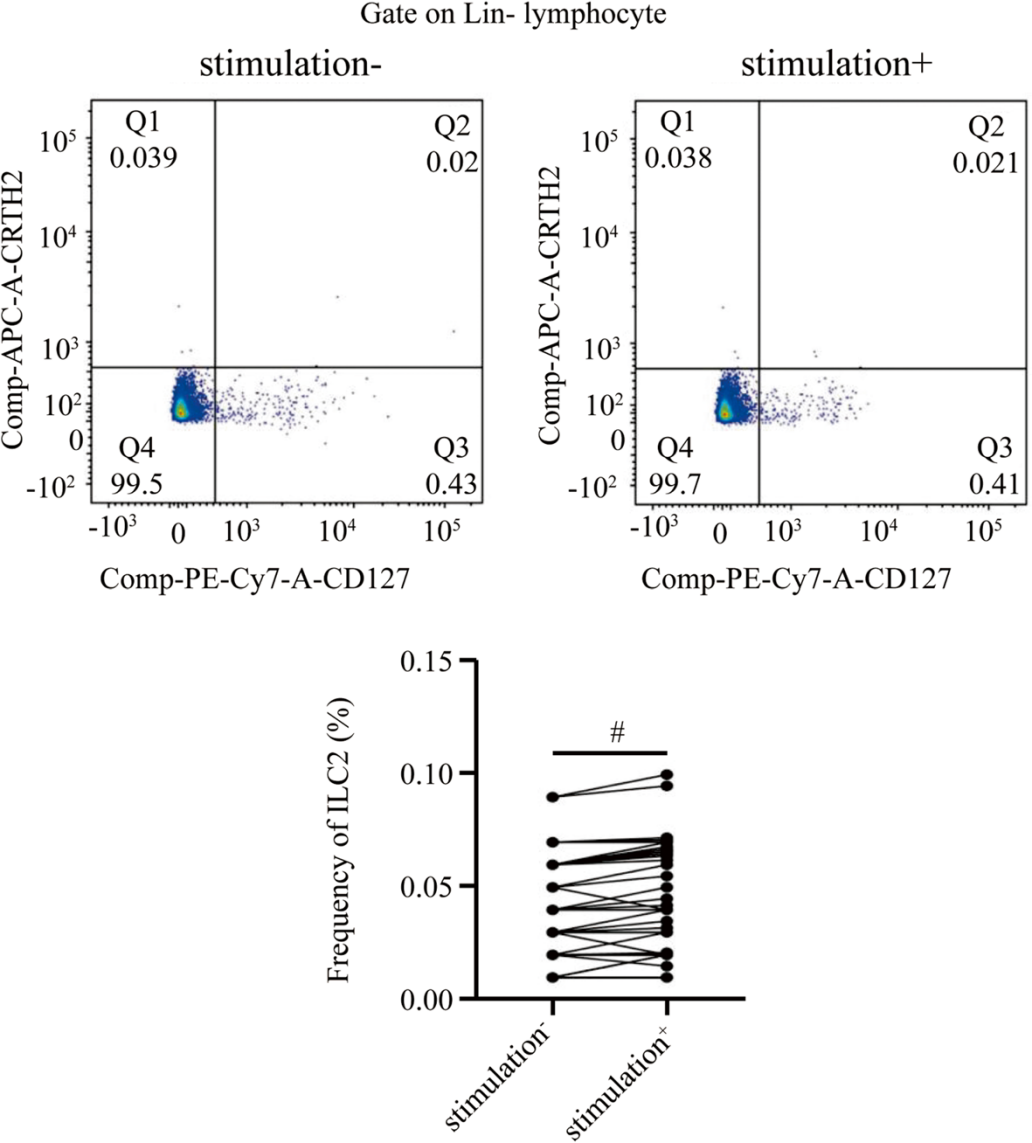
After stimulation with HDM (20 μg/mL) and stimulating factors, no significant change of ILC2 frequency in PBMCs from SLIT group. Two-year SLIT decreased the ILC2 milieu activation ability in AR donor. #P > 0.05. HDM, house dust mite; ILC2, type 2 innate lymphoid cells; PBMCs, peripheral blood mononuclear cells; SLIT, sublingual immunotherapy; ILC2, type 2 innate lymphoid cells; AR, allergic rhinitis

**Fig. 4:**
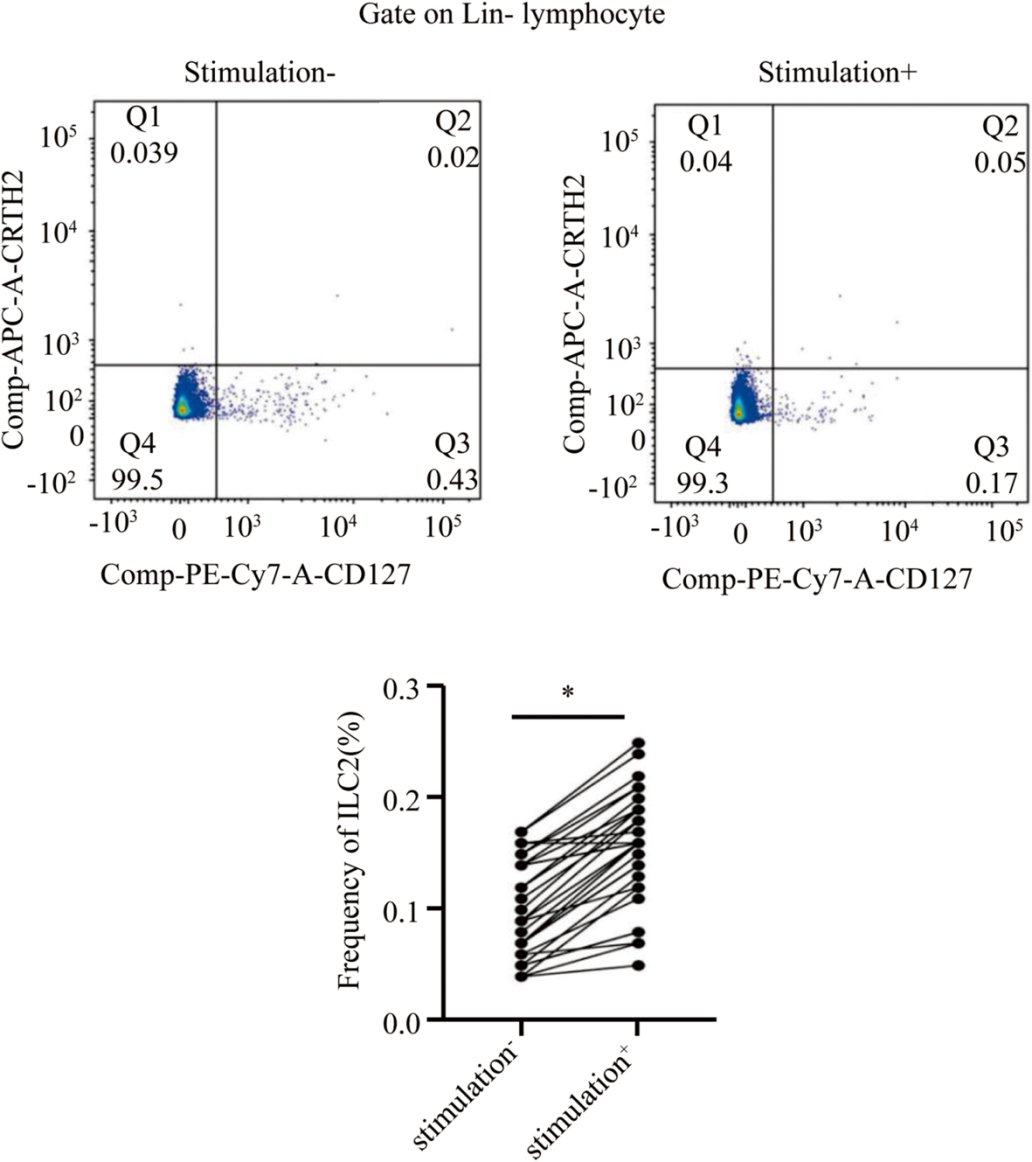
After stimulation with HDM (20 μg/mL) and stimulating factors, significant upregulation of ILC2 frequency in PBMCs from the control group. *P < 0.05. HDM, house dust mite; ILC2, type 2 innate lymphoid cells; PBMCs, peripheral blood mononuclear cells

**Fig. 5:**
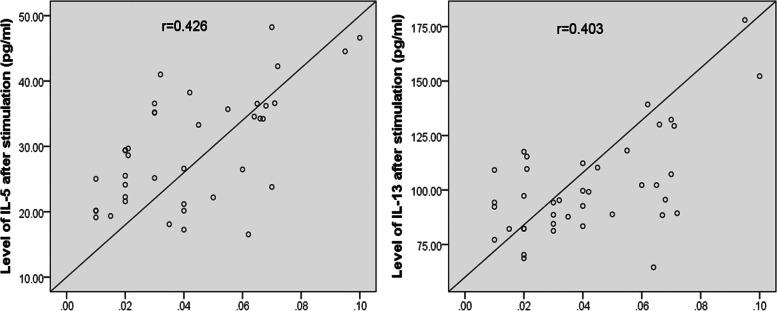
Positive correlation between levels of ILC2 and cytokines (IL-5/IL-13) after stimulation in the control group after 2-year treatment. ILC2, type 2 innate lymphoid cells; IL, interleukin

Both ILC2 frequency and ILC2-related cytokine (IL-5/IL-13) production in PBMCs were significantly inhibited in the SLIT group after 2-year SLIT treatment (ILC2: 0.040 ± 0.022 vs 0.043 ± 0.024, *P* = 0.601; IL-5: 26.63 ± 7.66 pg/mL vs 29.57 ± 8.86 pg/mL, *P* = 0.131; IL-13: 94.36 ± 23.15 pg/mL vs 102.81 ± 23.12 pg/mL, *P* = 0.120) (Figs. 3 and S6). However, the same indicator levels were significantly changed after stimulation in the control group after 2-year treatment (ILC2: 0.104 ± 0.043 vs 0.161 ± 0.049, IL-5: 60.61 ± 10.56 pg/mL*vs* 91.29 ± 12.39 pg/mL, IL-13: 143.27 ± 21.31 pg/mL vs 195.65 ± 29.81 pg/mL; *P*<0.05) (Figs. 4 and S7). Furthermore, a positive correlation was found between the levels of ILC2 and cytokines (IL-5/IL-13) after stimulation in the control group after 2-year treatment (*P* < 0.01) (Fig. 5).

## Discussion

SLIT, as the best treatment with the ability to modify the course of allergic disease, has been recommended as the first-line therapy for AR by several guidelines lately [3, 18]. The mechanism of its treatment to AR may largely be attributed to its inhibition of Th2 reaction, and the present study also found that inhibition of Th2 response-related cytokine (IL-5/IL- 13) expression was observed in the SLIT group after 1- and 2-year SLIT, which is consistent with the previous study results [13, 19]. Combined with the significantly decreased T5SS score after treatment in the SLIT group, the results confirmed the successful immunomodulatory function of SLIT in Chinese pediatric patients with AR. As a subpopulation of innate lymphoid cells, ILC2’s elevated expression in AR was often observed in previous studies, and it could induce an abnormally activated Th2 response and eosinophil recruitment after allergen stimulation in AR [20, 21]. Moreover, the severity of disease is related to the level of ILC2 in pediatric patients with AR, which further confirmed ILC2’s role in the pathogenesis of AR [6]. Since SLIT could inhibit the Th2 response and ILC2 has similar functions to Th2, we hypothesized that ILC2 may be affected by SLIT, and the changes in the ILC2 milieu (ILC2 cells, ILC2-related inflammatory factor) during SLIT may be involved in the therapeutic mechanism of SLIT. Our data showed that both ILC2 frequency and their transcription factor levels were decreased significantly in patients with AR 1 year after SLIT, and this trend was maintained for at least 2 years. These results suggest that SLIT could inhibit the abnormal expression of ILC2, and that the reducing level of ILC2 was positively correlated with the levels of Th2-related cytokine in the serum and nasal lavage, transcription factors, and T5SS score in patients with AR after 1- and 2-year SLIT in the present study, which confirmed that the inhibition of Th2 response and improvement of symptoms in AR by SLIT are possibly attributed to the regulation of ILC2 by SLIT. However, we only found the change and correlation from nasal ECP level, and the serum level of ECP did not change after SLIT; these results are consistent with those of previous studies [13, 22], which suggested that SLIT may have a more effective improvement in the nasal local immune environment in AR after 1 or 2 years. The difference between local and systemic circulation may be due to the time, dose, and individual responsiveness to drugs, and the particular mechanism needs to be further studied.

To further determine the effect of SLIT on the ILC2 milieu, the present study stimulated PBMCs from patients after 1- and 2-year SLIT treatment by HDM and ILC2-stimulating factor (TSLP, IL-33, IL-25) and then detected the changes in ILC2 and related cytokines. The data showed that after 2-year SLIT treatment, HDM and stimulating factors did not induce significant activation of the ILC2 milieu, such as increased ILC2 frequency and high expressed cytokine levels in the SLIT group. However, there was an opposite result in the control group. In this group, the intervention led to a significant increase in the levels of ILC2 milieu-related indicators, and a positive correlation was observed between the ILC2 frequency and expression of inflammatory cytokines (IL-5/IL-13). These data clarified the regulatory effect of SLIT on the ILC2 milieu in PBMCs and the role of ILC2 in the regulation of Th2 cytokines in AR. The ability of ILC2 to promote Th2 cytokine production in PBMCs was significantly reduced after 2-year SLIT, suggesting that the regulatory function of SLIT on ILC2 is one of the pathways to improve Th2 bias in immunotherapy.

Children with AR often experience sinusitis, asthma, or other related concomitant diseases [23, 24]. The expression of ILC2 may be influenced by all these illnesses; thus, further studies are needed to clarify the role and regulatory mechanism of ILC2 in AR associated with various diseases.

In conclusion, the data of the present study indicated that expression of ILC2 milieu-related indicators decreased after SLIT, and these low expression levels were maintained for at least 2 years. The reduced activation capacity of the ILC2 milieu is related to SLIT. These data suggest that ILC2s may be involved in SLIT, which partly explains the mechanism by which SLIT reduces the Th2 reaction. However, our study offers only a preliminary investigation of the role of ILC2 in SLIT. The sample size of present study is small, and the changes in immunity over a longer period after the end of treatment have not been observed. In the future, it is necessary to further study the specific signal pathway mechanism of SLIT on the changes of ILC2 cells, so as to further elucidate the therapeutic mechanism. Moreover, with further research, the changes of ILC2 might be considered as a potential predictor of SLIT curative effect evaluation.

## Supplementary Information

**Additional file 1 **Figure S1. After completing the 1- and 2-year treatment period, the significant down-regulation of ILC2-related transcription factors (GATA3, RORα) in PBMCs from AR donor. *P < 0.05, #*P* > 0.05. (a): SLIT group; (b): Control group. ILC2, type 2 innate lymphoid cells; GATA3, GATA binding protein 3; RORα, retinoic acid-related orphan receptor α; PBMCs, peripheral blood mononuclear cells; AR, allergic rhinitis; SLIT, sublingual immunotherapy

**Additional file 2 **Figure S2. After completing the 1- and 2-year treatment period, the significant downregulation of ILC2-related cytokines (IL-5/IL- 13) in nasal lavage and serum from AR donor. *P < 0.05, #*P* > 0.05. (a): SLIT group; (b): Control groupILC2, type 2 innate lymphoid cells; IL, interleukin; AR, allergic rhinitis; SLIT, sublingual immunotherapy

**Additional file 3.** Figure S3. After completing the 1- and 2-year treatment period, the significant downregulation of ECP in nasal lavage from AR donor. *P < 0.05, #P > 0.05. (a): SLIT group; (b): Control group. ECP, eosinophil cationic protein; AR, allergic rhinitis; SLIT, sublingual immunotherapy

**Additional file 4.** Figure S4. Positive correlations between the levels of transcription factors levels (GATA3, RORα) and ILC2 frequency in the SLIT group after 1- and 2-year treatment. (a): After 1-year treatment; (b): After 2-year treatment. GATA3, GATA binding protein 3; RORα, retinoic acid-related orphan receptor α; ILC2, type 2 innate lymphoid cells; SLIT, sublingual immunotherapy

**Additional file 5.** Figure S5. Positive correlations between the nasal level of ECP, T5SS score and ILC2 frequency in the SLIT group after 1- and 2-year treatment. (a): After 1-year treatment; (b): After 2-year treatment. ECP, eosinophil cationic protein; T5SS, Total 5 Symptom Score; ILC2, type 2 innate lymphoid cells

**Additional file 6 **Figure S6. After stimulation with HDM and stimulating factors, no significant change of ILC2-related cytokines (IL-5/IL-13) in PBMCs from the SLIT group. Two-year SLIT decreased the ILC2 milieu activation ability in AR. donor. #*P* > 0.05. HDM, house dust mite; ILC2, type 2 innate lymphoid cells; IL, interleukin; PBMCs, peripheral blood mononuclear cells; SLIT, sublingual immunotherapy; AR, allergic rhinitis

**Additional file 7 **Figure S7. After stimulation with HDM (20 μg/mL) and stimulating factors, significant upregulation of ILC2-related cytokines (IL-5/IL-13) in PBMCs from the control group. *P < 0.05. HDM, house dust mite; ILC2, type 2 innate lymphoid cells; IL, interleukin; PBMCs, peripheral blood mononuclear cells

## Data Availability

Datasets used or analyzed during the current study are available from the corresponding author on reasonable request.

## Abbreviations

SLIT: Sublingual immunotherapy
AR: Allergic rhinitis
HDM: House dust mite
ILC2: Type 2 innate lymphoid cells
IL: Interleukin
PBMCs: Peripheral blood mononuclear cells
ELISA: Enzyme-linked immunosorbent assay
