# Supplementary figures and images for: Changes in type 2 innate lymphoid cells and serum cytokines in sublingual immunotherapy in pediatric patients with allergic rhinitis
Source: BMC Pediatr. 2023 Jan 9;23:13. doi: 10.1186/s12887-022-03788-z (PMC9827662; doi:10.1186/s12887-022-03788-z)

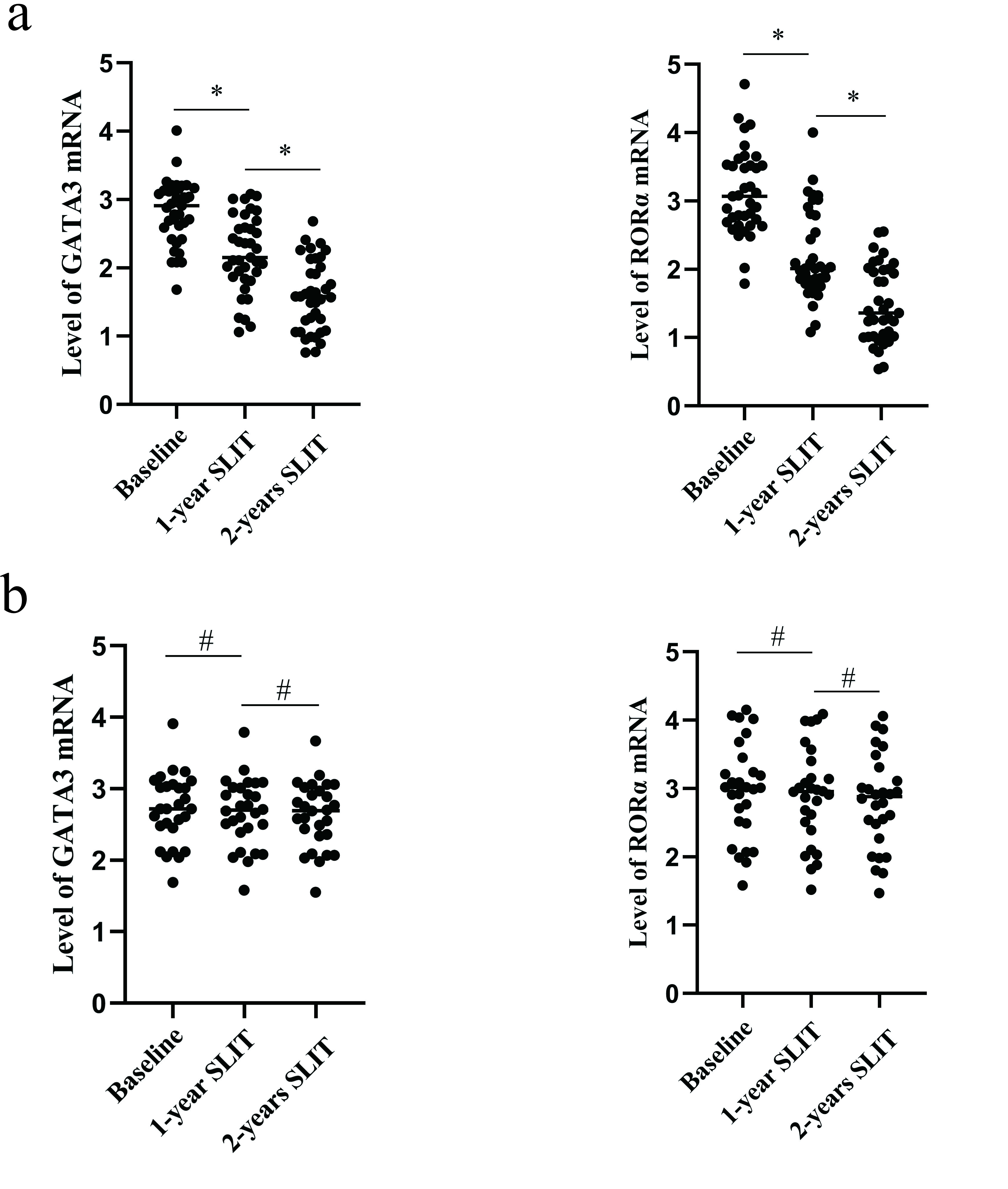

Supplement: Supplementary file 1 — Additional file 1 Figure S1. After completing the 1- and 2-year treatment period, the significant down-regulation of ILC2-related transcription factors (GATA3, RORα) in PBMCs from AR donor. *P < 0.05, #P > 0.05. (a): SLIT group; (b): Control group. ILC2, type 2 innate lymphoid cells; GATA3, GATA binding protein 3; RORα, retinoic acid-related orphan receptor α; PBMCs, peripheral blood mononuclear cells; AR, allergic rhinitis; SLIT, sublingual immunotherapy [file 12887_2022_3788_MOESM1_ESM.tif]

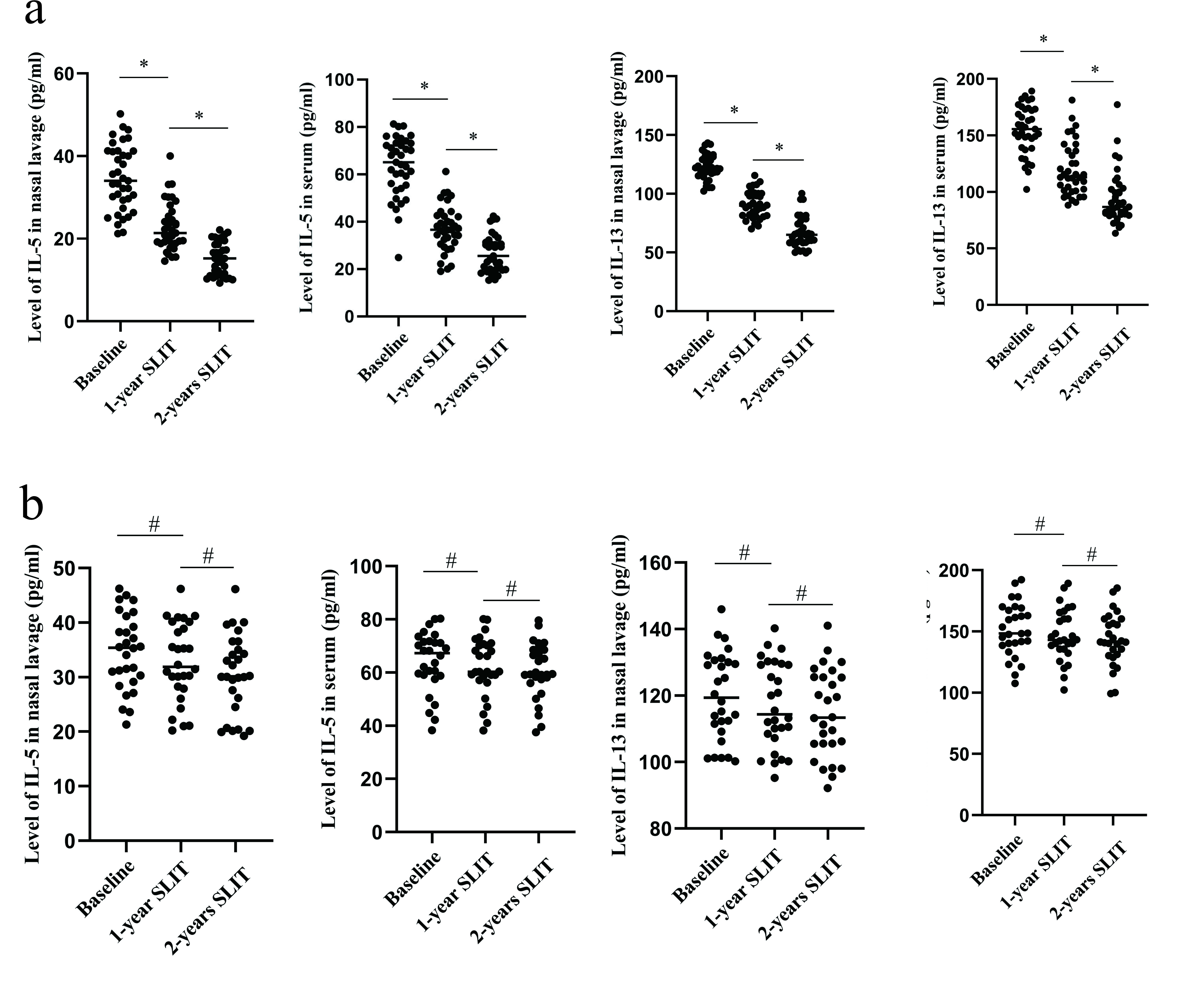

Supplement: Supplementary file 2 — Additional file 2 Figure S2. After completing the 1- and 2-year treatment period, the significant downregulation of ILC2-related cytokines (IL-5/IL- 13) in nasal lavage and serum from AR donor. *P < 0.05, #P > 0.05. (a): SLIT group; (b): Control groupILC2, type 2 innate lymphoid cells; IL, interleukin; AR, allergic rhinitis; SLIT, sublingual immunotherapy [file 12887_2022_3788_MOESM2_ESM.tif]

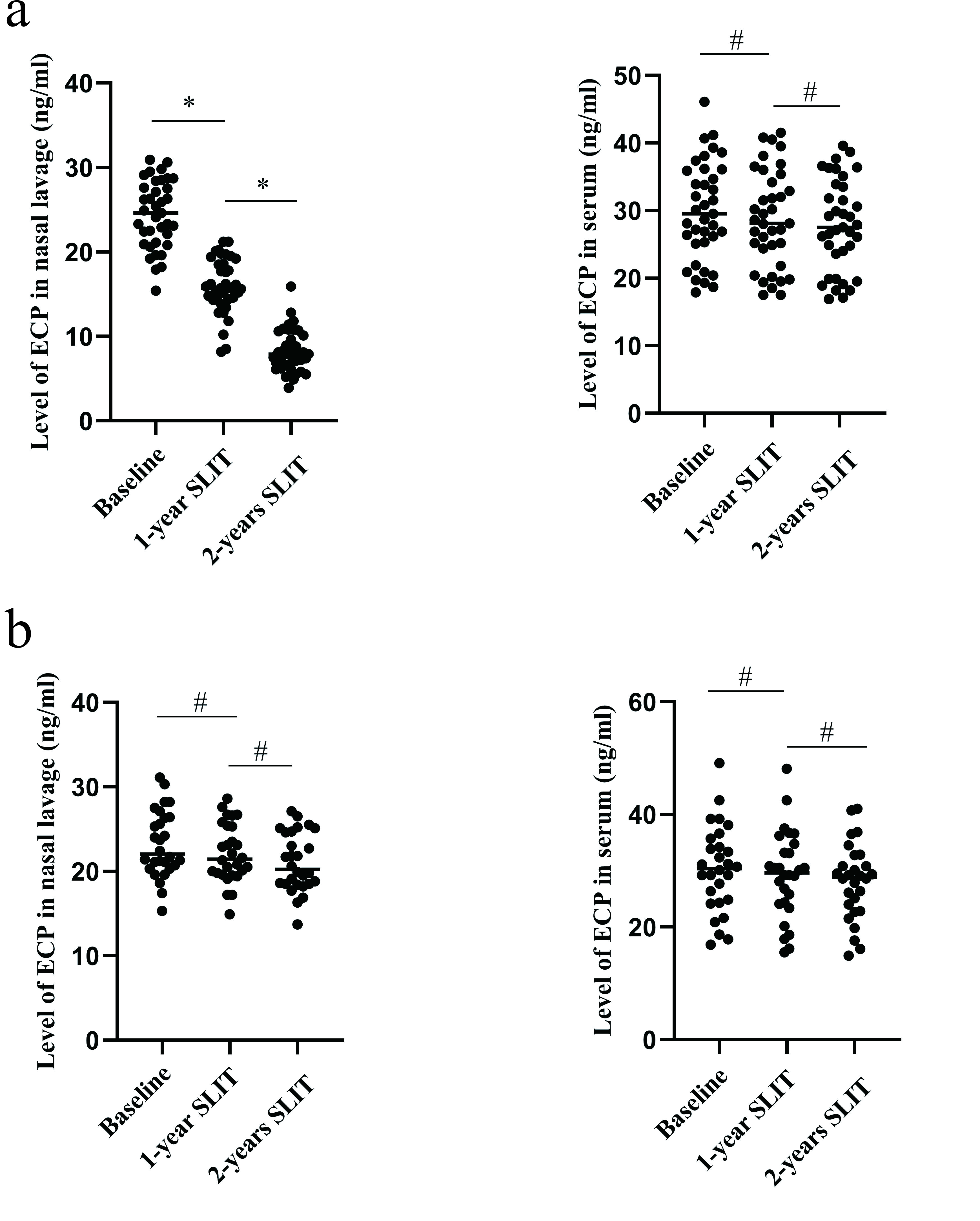

Supplement: Supplementary file 3 — Additional file 3. Figure S3. After completing the 1- and 2-year treatment period, the significant downregulation of ECP in nasal lavage from AR donor. *P < 0.05, #P > 0.05. (a): SLIT group; (b): Control group. ECP, eosinophil cationic protein; AR, allergic rhinitis; SLIT, sublingual immunotherapy [file 12887_2022_3788_MOESM3_ESM.tif]

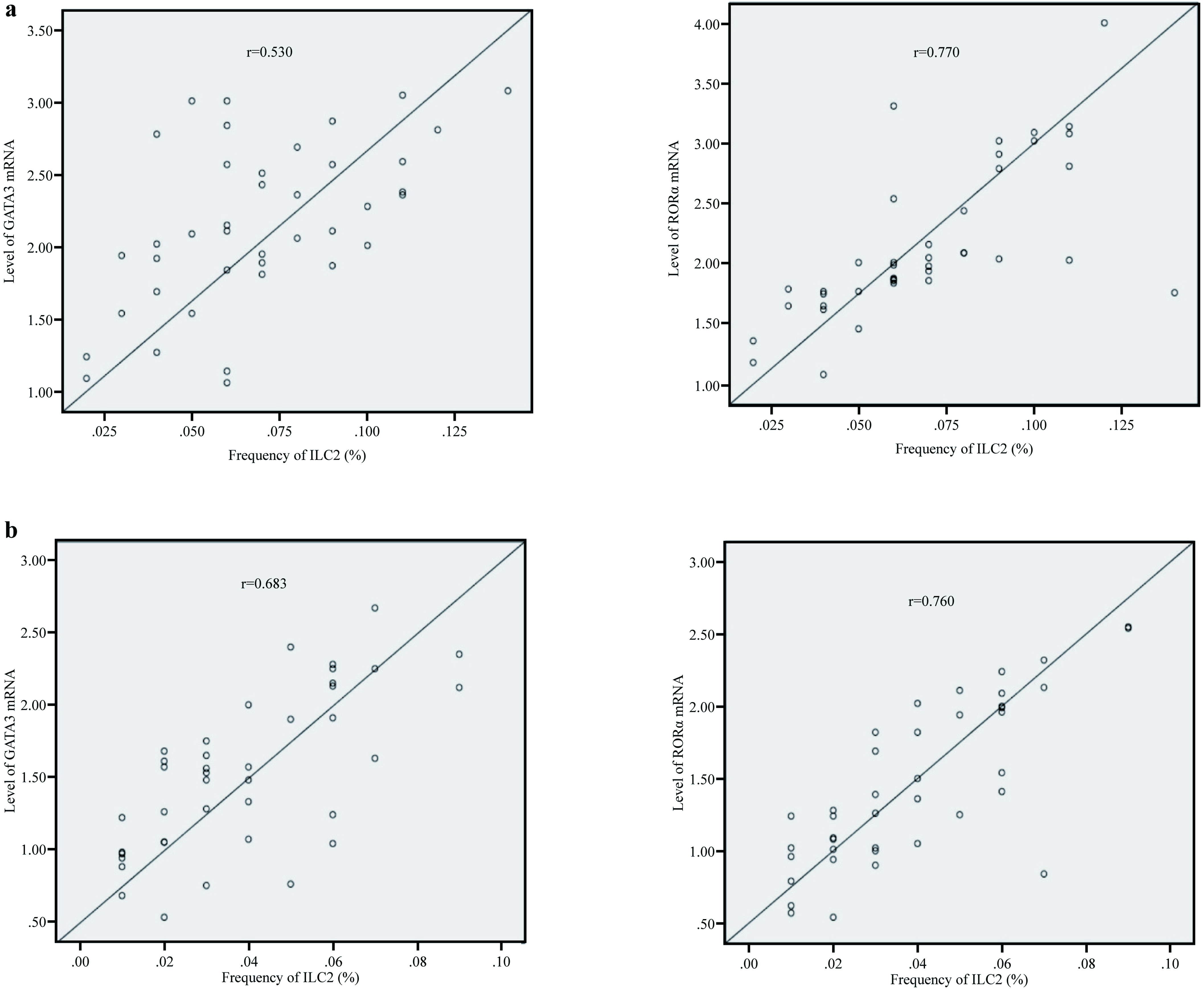

Supplement: Supplementary file 4 — Additional file 4. Figure S4. Positive correlations between the levels of transcription factors levels (GATA3, RORα) and ILC2 frequency in the SLIT group after 1- and 2-year treatment. (a): After 1-year treatment; (b): After 2-year treatment. GATA3, GATA binding protein 3; RORα, retinoic acid-related orphan receptor α; ILC2, type 2 innate lymphoid cells; SLIT, sublingual immunotherapy [file 12887_2022_3788_MOESM4_ESM.tif]

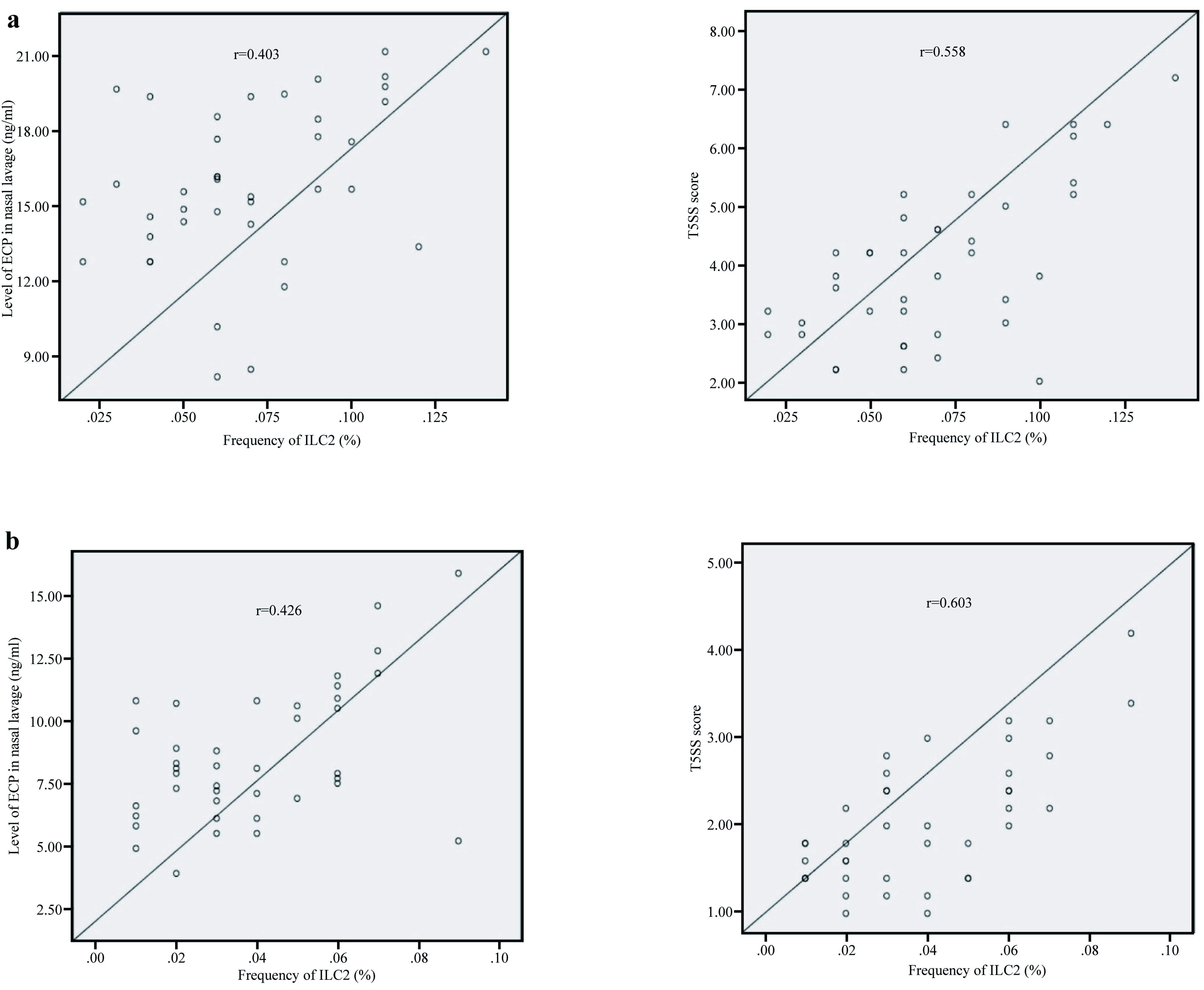

Supplement: Supplementary file 5 — Additional file 5. Figure S5. Positive correlations between the nasal level of ECP, T5SS score and ILC2 frequency in the SLIT group after 1- and 2-year treatment. (a): After 1-year treatment; (b): After 2-year treatment. ECP, eosinophil cationic protein; T5SS, Total 5 Symptom Score; ILC2, type 2 innate lymphoid cells [file 12887_2022_3788_MOESM5_ESM.tif]

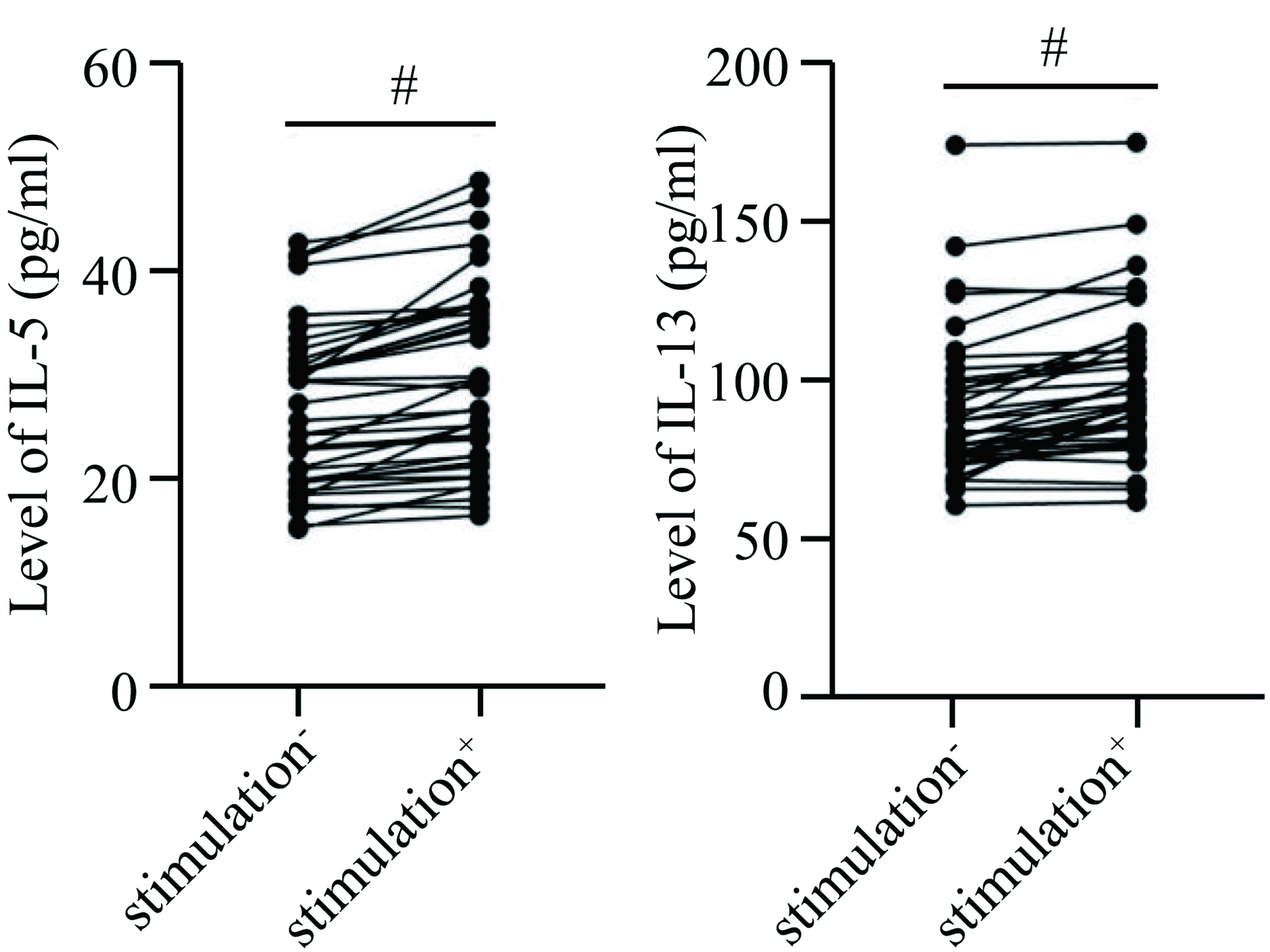

Supplement: Supplementary file 6 — Additional file 6 Figure S6. After stimulation with HDM and stimulating factors, no significant change of ILC2-related cytokines (IL-5/IL-13) in PBMCs from the SLIT group. Two-year SLIT decreased the ILC2 milieu activation ability in AR. donor. #P > 0.05. HDM, house dust mite; ILC2, type 2 innate lymphoid cells; IL, interleukin; PBMCs, peripheral blood mononuclear cells; SLIT, sublingual immunotherapy; AR, allergic rhinitis [file 12887_2022_3788_MOESM6_ESM.tif]

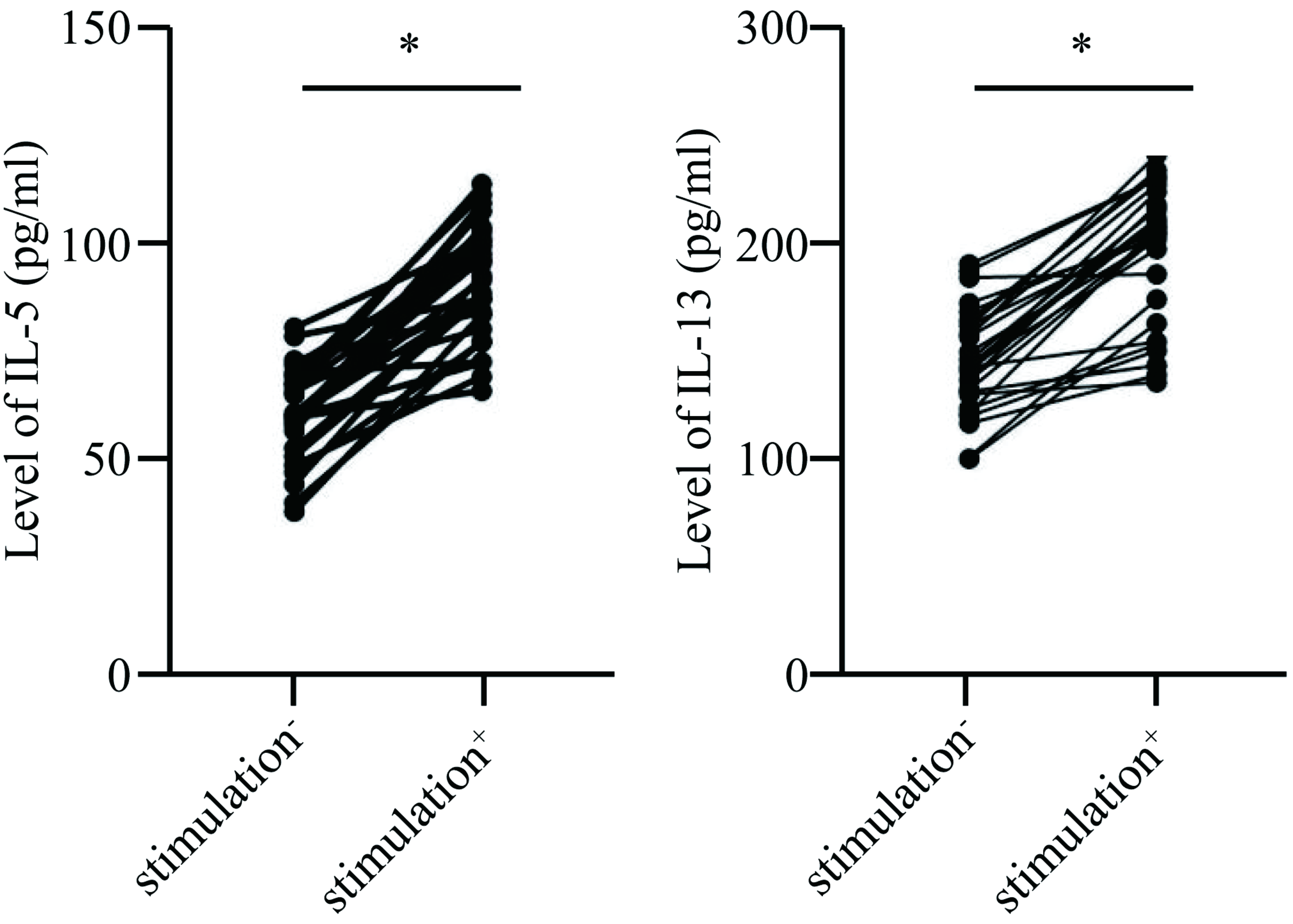

Supplement: Supplementary file 7 — Additional file 7 Figure S7. After stimulation with HDM (20 μg/mL) and stimulating factors, significant upregulation of ILC2-related cytokines (IL-5/IL-13) in PBMCs from the control group. *P < 0.05. HDM, house dust mite; ILC2, type 2 innate lymphoid cells; IL, interleukin; PBMCs, peripheral blood mononuclear cells [file 12887_2022_3788_MOESM7_ESM.tif]
